# The Differential Diagnosis of Hypopigmented Mycosis Fungoides and Vitiligo With Reflectance Confocal Microscopy: A Preliminary Study

**DOI:** 10.3389/fmed.2020.609404

**Published:** 2021-01-11

**Authors:** Huaxu Liu, Leilei Wang, Yan Lin, Xiaofeng Shan, Min Gao

**Affiliations:** ^1^Shandong Provincial Institute of Dermatology and Venereology, Shandong First Medical University & Shandong Academy of Medical Sciences, Jinan, China; ^2^Shandong Cancer Hospital and Institute, Shandong First Medical University & Shandong Academy of Medical Sciences, Jinan, China

**Keywords:** imaging, reflectance confocal microscopy (RCM), detection, vitiligo, hypo-pigmented mycosis fungoides, differential daignosis

## Abstract

**Objective:** To investigate the role of reflectance confocal microscopy (RCM) in the differential diagnosis of hypopigmented mycosis fungoides (HMF) and vitiligo.

**Methods:** Cases with persistent hypopigmented patches, suspicious of early stage vitiligo, or HMF were imaged with RCM. The melanin contents and inflammatory conditions of the epidermis and superficial dermis of the lesions were compared with the same layers of the adjacent skin, and then, the imaged lesions were biopsied and analyzed by histology.

**Results:** 15 cases were enrolled in this study, and based on the RCM findings, there was just slight or moderate reduction of melanin but no melanin absence in the basal cell layer of HMF lesions. The finding of monomorphous weakly refractile, oval to round cells on the basis of vesicle-like dark space was clearly elucidated in the epidermis of the lesions by RCM, which indicates the Pautrier's microabscesses on histopathology. Among those 15 cases, 13 cases were identified as HMF, and the other two cases were vitiligo, based on RCM findings, which were confirmed by histology analysis.

**Conclusions:** The RCM findings correlated well with histology results in the screening of HMF, which indicates the RCM is an important tool in the early detection and differential diagnosis of HMF.

## Introduction

Mycosis fungoides (MF), the most common primary cutaneous T-cell lymphoma, is a neoplastic disease characterized by classical non-infiltrated lesions (patches), plaques, tumors, and erythrodermic stages (1). Several distinct clinical forms of MF have been described, among which, the granulomatous, pustular, purpuric, hyperkeratotic and verrucous, bullous, invisible, and hypopigmented variants of the disease were included (1). The hypopigmented mycosis fungoides (HMF) was first described by (2, 3). It is an atypical and often misdiagnosed variant of MF characterized by persistent hypopigmented-to-achromic patches, with a vitiligo-like aspect, which is mainly distributed on the trunk and proximal portions of the extremities (1, 3). Unlike conventional MF, which is regarded as a disease most commonly found in the fifth to sixth decades of life, HMF most commonly affects the pediatric population, especially in Asians (1, 3). Clinically, the diagnosis of HMF is commonly delayed, which may have a potential negative effect on the treatment and prognosis because it is rare, and it may resemble early vitiligo and other hypopigmentary skin disorders (4–6).

Reflectance confocal microscopy (RCM) is a non-invasive imaging technique that provides high-resolution cellular imaging of the epidermis and superficial dermis (7), which has been used in the diagnosis and differential diagnosis of skin tumors and inflammatory diseases for more than two decades with high sensitivity and specificity (8). Agero et al. (9) first investigated the characteristics of MF in the imaging of RCM. The Pautrier's microabscesses in epidermis on histopathology correlated with the vesicle-like dark space filled with collections of monomorphous weakly refractile oval to round cells in epidermis on the RCM images. Other studies (10) verified the correlation of RCM image and histology analysis in the diagnosis of MF.

In this study, we aimed to investigate the role of RCM in the early detection and differential diagnosis of HMF.

## Materials and Methods

The study was approved by the Ethics Committee of Shandong Provincial Institute of Dermatology and Venereology. The cases with persistent round-to-oval hypopigmentation with mild scales or not, which distributed on the trunk, arms, and legs, wood lamp examination (±) and clinically suspicious of HMF and early vitiligo, were enrolled in the study. Cases with comparatively typical vitiligo lesions were not included in the study.

After written informed consent obtained and the related history recorded, the clinical pictures were taken and the hypopigmentary lesions and adjacent normal skin were imaged with RCM. *In-vivo* RCM imaging was performed with a commercially available, near-infrared, reflectance mode confocal microscope (Vivascope 1500; Lucid Inc., Rochester, NY, USA). A detailed description of the technique and the device used has been published previously (7, 8). At least three areas of hypopigmented lesions were imaged for each case to compare the changes with the perilesional normal skin. The single (0.5^*^0.5 mm) or mosaic RCM images (2^*^2 mm, or 3^*^3 mm) were captured or saved. Then one of the lesions imaged were suggested to be biopsied and analyzed histologically. The excisions were fixed in formalin and embedded in paraffin. After routine processing, slides were stained with hematoxylin and eosin (H–E) and further stained with immune-chemistry methods to detect the changes of CD4 and CD8 molecules.

Based on the previous studies, the RCM features of the lesions were analyzed layer by layer and correlated to the findings of histology analysis.

## Results

In total, 15 cases with RCM and histology results were enrolled in the study. Among those enrolled 15 cases, 13 cases were diagnosed as HMF, while two cases as vitiligo, based on our RCM examination, and was demonstrated by histology results, which showed the excellent correlation between RCM and histology findings.

**Figure 1:**
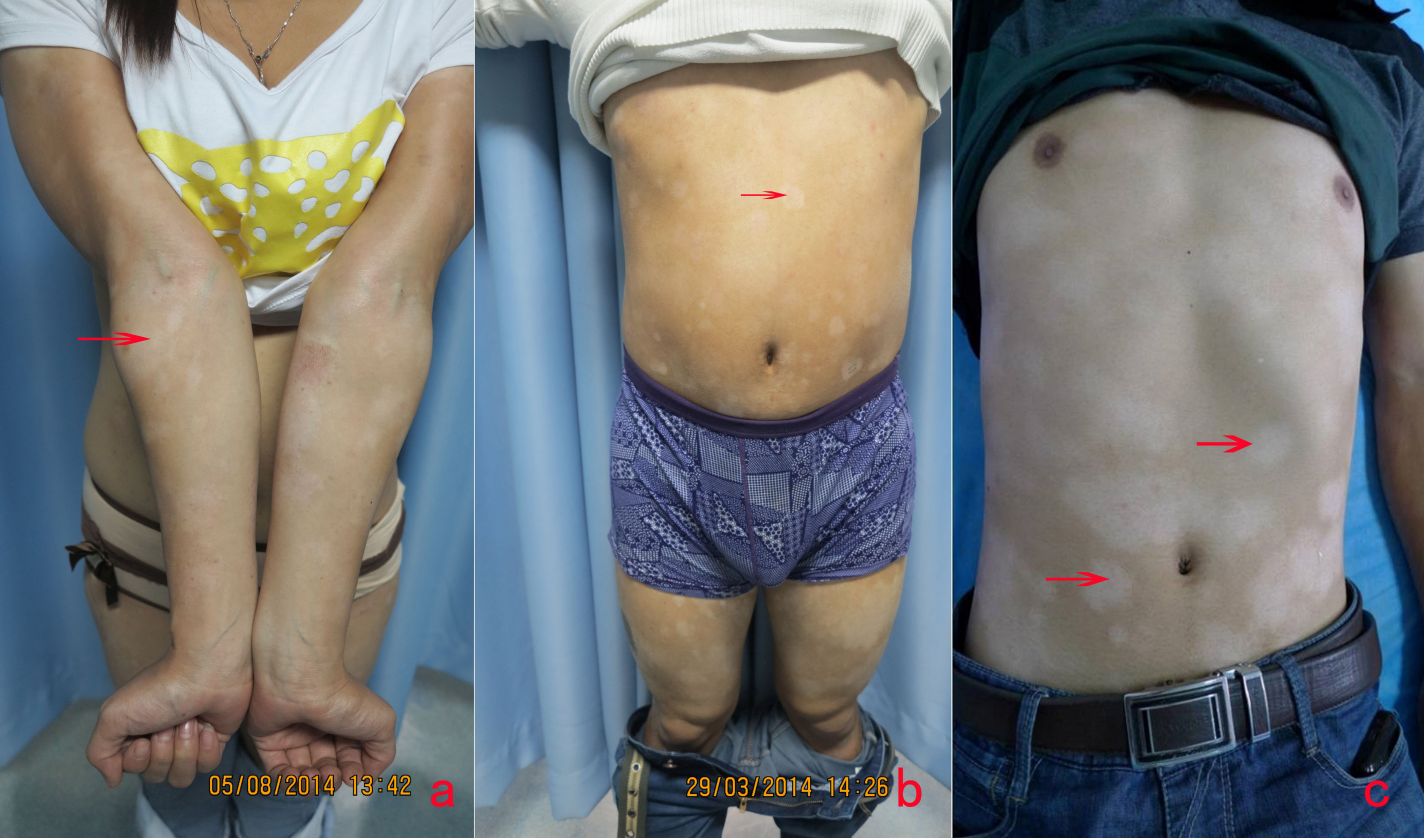
The clinical image of three cases of HMF. An 18-year old woman with the hypopigmented patches for 2 years on arms **(a)** and legs, and a 25-year-old **(b)** and 19-year-old man **(c)**, with generalized lesions on bodies and limbs. The hypopigmentary patches were 2–5 cm in diameter, slightly scaly, with no infiltration (arrows).

Five of the 15 cases were male, 10 were female, and the average age of the 15 cases was 19.2. The average duration of evolution of the lesions was 26.9 months. The details of the enrolled cases are listed in Table 1. Clinically, the persistent, round-to-oval hypopigmentation with mild scales or not were distributed on the trunk, arms, and legs (Figures 1a–c).

**Table 1:** The details of the included cases.

**Case**	**Age**	**Sex**	**Main distribution**	**Duration of evolution (month)**	**Previous tretments**	**RCM findings**	**Pathology findings**
1	15	M	Trunk and legs	36	Topical steroids	+	+
2	20	F	Trunk and legs	40	none	+	+
3	14	F	Trunk, arms, and legs	32	Topical steroids	+	+
4	31	F	Trunk and legs	72	none	+	+
5	21	F	Trunk and arms	24	Topical steroids	+	+
6	15	M	Trunk, arms, and legs	12	Topical steroids	+	+
7	22	F	Trunk, arms, and legs	26	Topical steroids	+	+
8	21	M	Trunk, arms, and legs	31	Topical steroids	+	+
9	19	F	Trunk, arms, and legs	37	Topical steroids	+	+
10	17	F	Trunk and legs	16	Topical steroids	+	+
11	18	F	Trunk, arms, and legs	22	Topical steroids	+	+
12	20	M	Trunk and arms	28	Topical steroids	+	+
13	19	F	Trunk, arms, and legs	20	none	+	+
14	16	M	Trunk	5	Topical steroids	Absence of melanin	vitiligo
15	20	F	Trunk	3	Topical steroids	Absence of melanin	vitiligo
Mean	19.2			26.9			

*(1) The lesions were mainly located on the trunk and limbs. (2) The duration of evolution was calculated from the early onset of hypopigmented patches, according to the patients' medical histories. (3) The RCM findings was the slight melanin reduction, but not melanin absence in the basal cell layer, and the monomorphous weakly refractile, oval-to-round cells in epidermis and weakly refractive cells with a round-to-oval cellular contour scattered within the papillary dermis or inside dermal papillae rings were the positive findings (+). (4) The histology and immunochemistry findings to confirm those RCM features and diagnosed as HMF was the “+” in the table*.

**Figure 2:**
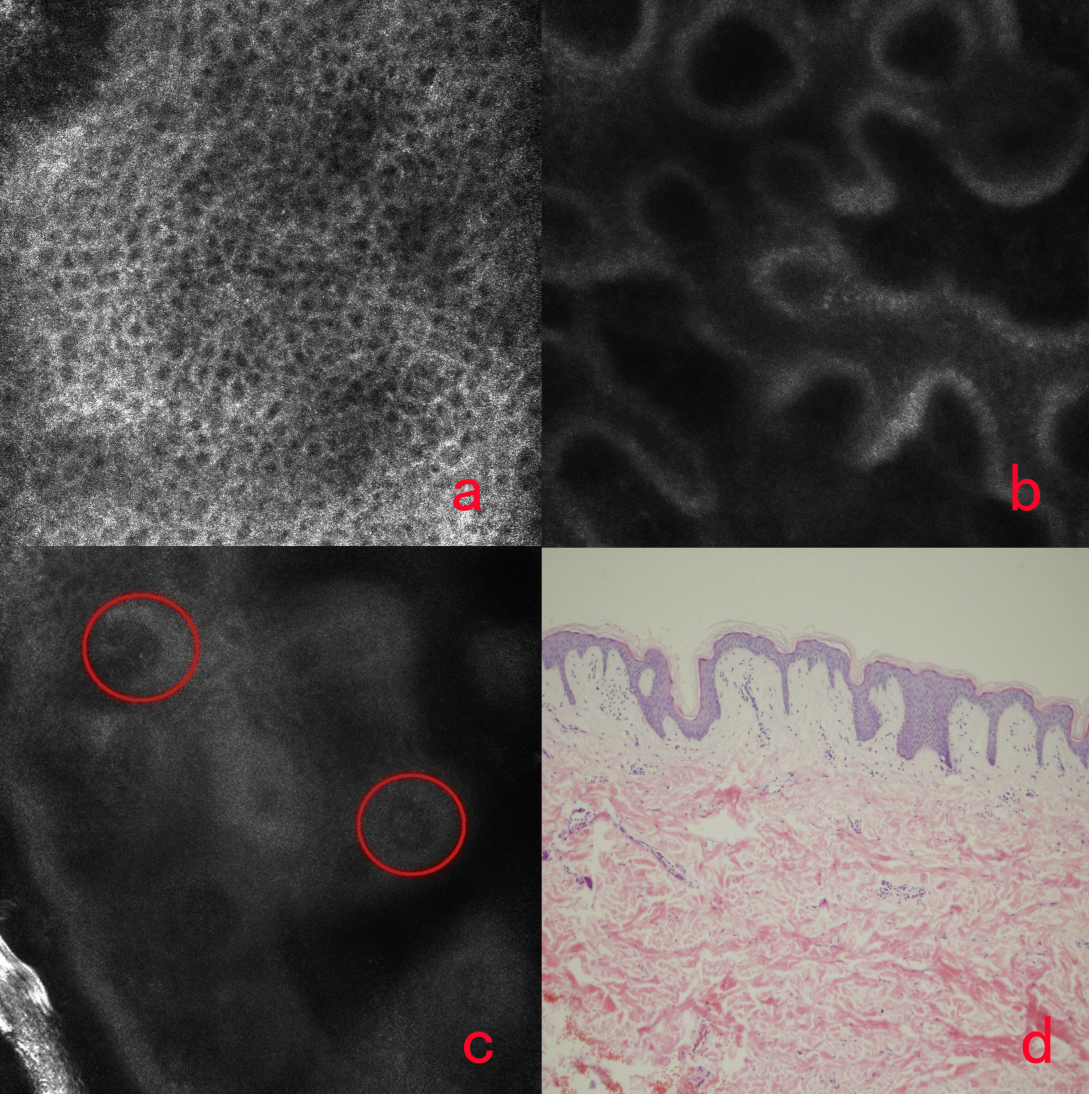
The honeycomb pattern of stratum spinosum **(a)**, the normal basal cell layer, the dermal-epidermal junction of vitiligo **(c)** and the histology of vitiligo. There was no inflammation in the regular arranged honeycomb pattern of stratum spinosum **(a)**. There was no melanin reduction in the basal cell layer of normal DEJ **(b)**. The significant reduction or melanin absence (circles) in the basal cell layer of DEJ of vitiligo **(c)**. There is no melanin in basal cell layer of vitiligo **(d)**.

When the normal skin was imaged with RCM, there were no significant inflammatory condition changes in honeycomb pattern stratum spinosum (Figure 2a) and no melanin changes in basal cell layer (Figure 2b). While for the lesions of vitiligo, the significant reduction of melanin contents or melanin absence was observed (Figure 2c) compared with the adjacent normal skin, and sometimes the slight infiltration of phagocytes and inflammatory cells in the superficial dermis was also recorded. The histology picture (Figure 2d) demonstrated the RCM findings.

**Figure 3:**
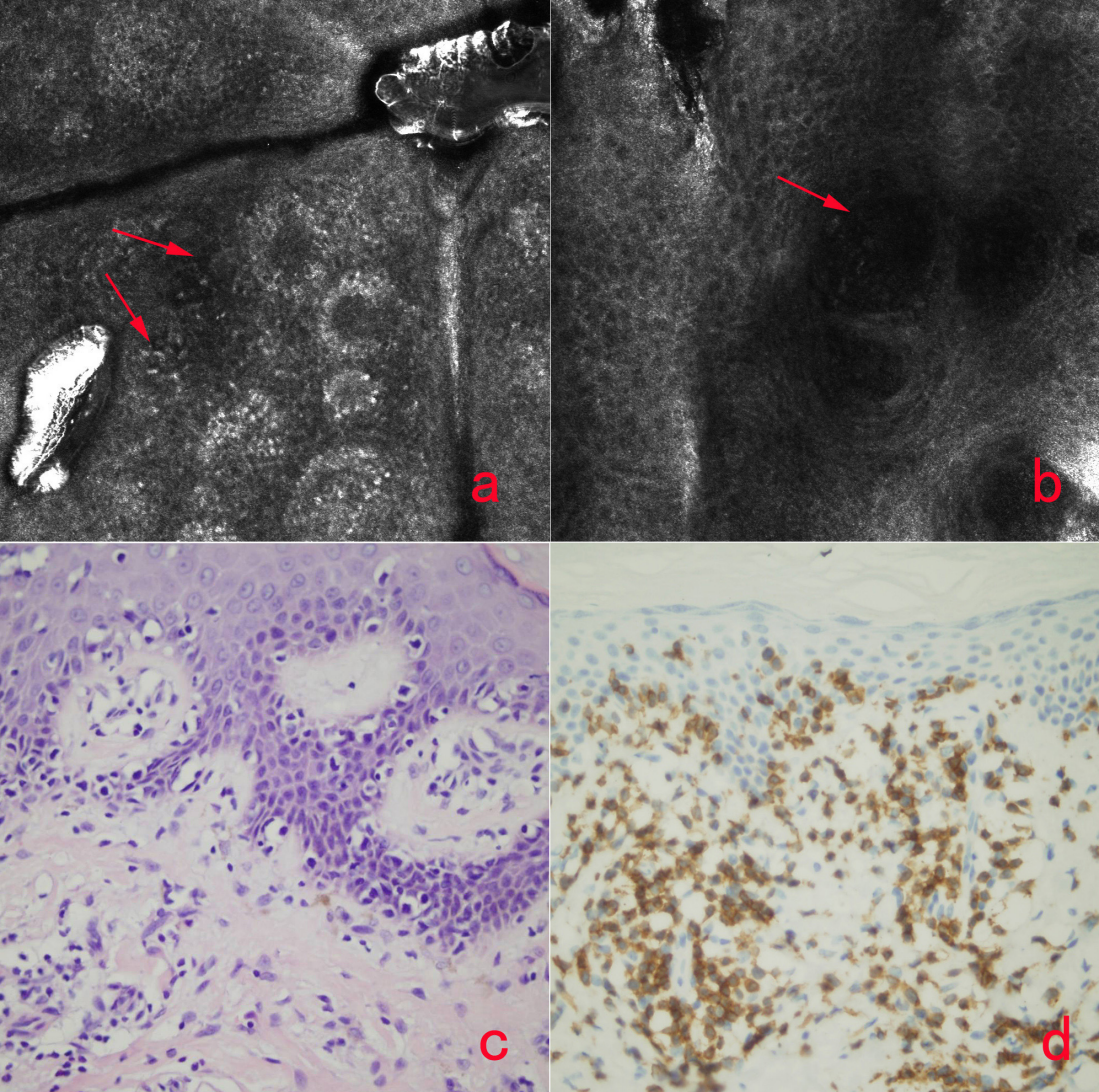
The RCM image of HMF **(a,b)**, the histology of HMF **(c)** and immunochemistry-stained image of HMF **(d)**. The monomorphous weakly refractile, oval-to-round cells (**a,b**, arrows) on the basis of vesicle-like dark spaces were clearly elucidated in epidermis of the lesions by RCM, which indicates the Pautrier's microabscesses on histopathology **(c)**. Immunohistochemical analysis **(d)** demonstrated the HMF was characterized by CD8+ T cells residing predominantly in the dermal papillae.

The HMF lesions showed different changes based on the RCM imaging, as there was only slight reduction of melanin contents but no melanin absence in the basal cell layer. And the other significant changes were founded in stratum spinosum. The finding of monomorphous weakly refractile, oval-to-round cells on the basis of vesicle-like dark space was clearly elucidated in epidermis of the lesions by RCM (Figures 3a,b), which indicates the Pautrier's microabscesses on histopathology (Figure 3c). And weakly reflective cells with a round-to-oval cellular contour scattered within the papillary dermis or inside dermal papillae rings in the lesions were also observed. Immunohistochemistry analysis (Figure 3d) demonstrated the HMF was characterized by CD8+ T cells residing predominantly in the dermal papillae. Thirteen cases with these findings by RCM were confirmed by pathology results.

## Discussion

RCM imaging non-invasively shows nuclear and cellular-level morphology in human skin *in vivo* (11, 12). Imaging is based on the detection of single back-scattered photons from the optical section, and contrast is due to the relative variations in refractive indices and sizes of organelles and microstructures (11). Melanin is the natural and strongest contrast in the skin (11, 12), which indicates the significant role of RCM in the imaging of pigmented skin disorders. The cellular changes of the lesion in epidermis and superficial dermis could be imaged and compared with that of the adjacent normal skin. Ardigo et al. (13) first reported the characteristics of vitiligo in confocal images, and other related reports (14) revealed RCM is useful in the diagnosis and differential diagnosis of vitiligo and other hypopigmentary skin disorders. As previously mentioned, RCM has also been reported to be used in the screening of MF and showed relatively specific characteristics in confocal images (9, 10, 15, 16). The migration of abnormal inflammatory cells in the epidermis, the disorder of the basal cell layer, and infiltration of mononuclear inflammatory cells in superficial dermis of MF lesions could be imaged with RCM (15).

The pathology features of HMF are similar to MF. And the histology changes of HMF and vitiligo were located in epidermis and superficial dermis,which was within the penetration depth of RCM.

Our study revealed the melanin contents in the basal cell layer is different based on the RCM findings. Significant melanin reduction or melanin absence was found in vitiligo, while there was a slight reduction of melanin but not melanin loss in HMF. As reported previously, hypopigmentation of HMF may arise from a defect in the transfer of melanosomes from melanocytes to keratinocytes, with reversal of this defect after treatment (4). On the contrary, the significant reduction or total absence of melonosome and melanocytes is the characteristic in the vitiligo lesion (6). The other difference was focused on the stratum spinosum and superficial dermis. The finding of monomorphous weakly refractile, oval-to-round cells on the basis of vesicle-like dark space was regarded as the features of HMF by RCM, while commonly there were no changes in this layer in vitiligo. And weakly refractive cells with a round-to-oval cellular contour scattered within the papillary dermis or inside dermal papillae rings in HMF were also different with that of vitiligo because these changes were the characteristics of HMF in histology level. RCM and histology results had excellent correlation.

Our study highly indicated the role of RCM in the early detection and differential diagnosis of HMF and vitiligo. However, more cases with long time follow-up study were required to confirm our findings.

## Data Availability Statement

The original contributions presented in the study are included in the article/supplementary materials, further inquiries can be directed to the corresponding author.

## Ethics Statement

The studies involving human participants were reviewed and approved by Ethics Committee of Shandong Provincial Institute of Dermatology and Venereology. Written informed consent to participate in this study was provided by the participants' legal guardian/next of kin. Written informed consent was obtained from the minor(s)' legal guardian/next of kin for the publication of any potentially identifiable images or data included in this article.

## Author Contributions

HL and MG designed the study and prepared the manuscript. YL and LW collected the clinical and RCM data. XS collected the histology data. All authors contributed to the article and approved the submitted version.

## Conflict of Interest

The authors declare that the research was conducted in the absence of any commercial or financial relationships that could be construed as a potential conflict of interest.
